# Insights into genomics of salt stress response in rice

**DOI:** 10.1186/1939-8433-6-27

**Published:** 2013-10-28

**Authors:** Kundan Kumar, Manu Kumar, Seong-Ryong Kim, Hojin Ryu, Yong-Gu Cho

**Affiliations:** Department of Biological Sciences, Birla Institute of Technology & Science, K. K. Birla Goa Campus, Goa 403726 India; Department of Life Science, Sogang University, Seoul, 121-742 Korea; Department of Life Science, Pohang University of Science & Technology, Pohang, Korea; Department of Crop Science, Chungbuk National University, Cheongju, 361-763 Korea

**Keywords:** Rice, Salt stress, Osmoprotectants, Signaling molecules, Transporters

## Abstract

Plants, as sessile organisms experience various abiotic stresses, which pose serious threat to crop production. Plants adapt to environmental stress by modulating their growth and development along with the various physiological and biochemical changes. This phenotypic plasticity is driven by the activation of specific genes encoding signal transduction, transcriptional regulation, ion transporters and metabolic pathways. Rice is an important staple food crop of nearly half of the world population and is well known to be a salt sensitive crop. The completion and enhanced annotations of rice genome sequence has provided the opportunity to study functional genomics of rice. Functional genomics aids in understanding the molecular and physiological basis to improve the salinity tolerance for sustainable rice production. Salt tolerant transgenic rice plants have been produced by incorporating various genes into rice. In this review we present the findings and investigations in the field of rice functional genomics that includes supporting genes and networks (ABA dependent and independent), osmoprotectants (proline, glycine betaine, trehalose, myo-inositol, and fructans), signaling molecules (Ca^2+^, abscisic acid, jasmonic acid, brassinosteroids) and transporters, regulating salt stress response in rice.

**Electronic supplementary material:**

The online version of this article (doi:10.1186/1939-8433-6-27) contains supplementary material, which is available to authorized users.

## Introduction

World agriculture faces a challenging task to produce 70% more food for an additional 2.3 billion people by 2050 (FAO 2009). The lower agriculture crop productivity is mostly attributed to various abiotic stresses, which is a major area of concern to cope with the increasing food requirements (Shanker and Venkateswarlu 2011). The major abiotic stresses includes high salinity, drought, cold, and heat negatively influence the survival, biomass production and yield of staple food crops which is a major threat to food security worldwide (Thakur et al. 2010; Mantri et al. 2012).

Among abiotic stresses, soil salinity is one of the most brutal environmental factors and a complex phenotypic and physiological phenomenon in plants imposing ion imbalance or disequilibrium, hyperionic and hyperosmotic stress, disrupting the overall metabolic activities and thus limiting the productivity of crop plants worldwide (Munns and Tester 2008). Worldwide more than 80 million hectares of irrigated land (representing 40% of total irrigated land) have already been damaged by salt (Xiong and Zhu 2001). Salt stress leads to severe inhibition of plant growth and development, membrane damages, ion imbalances due to Na^+^ and Cl^-^ accumulation, enhanced lipid peroxidation and increased production of reactive oxygen species like superoxide radicals, hydrogen peroxide and hydroxyl radicals. Area under salt stress is increasing due to many factors including climate change, rise in sea levels, excessive irrigation without proper drainage in inlands and underlying rocks rich in harmful salts etc. It is estimated that if current scenario of salinity stress would persist, there may be loss of 50% of present cultivated land for agriculture by 2050 (Wang et al. 2003).

Rice (*Oryza sativa* L.) is the world’s most important food crop and a primary source of food for more than half of the population. More than 90 per cent of the world’s rice is grown and consumed in Asia, where 60 percent of the earth’s people live. Salinity is the most common abiotic stress encountered by rice plants and classified as a salt sensitive crop in their early seedling stages (Lutts et al. 1995) and limit its productivity (Todaka et al. 2012). To improve the yield under salt stress condition, it is essential to understand the fundamental molecular mechanisms behind stress tolerance in plants. Salinity stress tolerance is a quantitative trait which is controlled by multiple genes (Chinnusamy et al. 2005). During the last two decades, number of genes conferring salt stress tolerance in plants have been isolated and they are involved in signal transduction and transcription regulation (Chinnusamy et al. 2006; Kumari et al. 2009), ion transporters (Verma et al. 2007; Singh et al. 2008; Uddin et al. 2008) and metabolic pathways (Sakamoto et al. 1998; Singla-Pareek et al. 2008). In the current review we try to focus on the salt responsive genes and genome networks, signal transduction, osmoprotectants and ion transporters involved in salinity stress response in rice.

## Review

### Salt stress tolerance-supportive genes and pathways in rice

**Figure 1:**
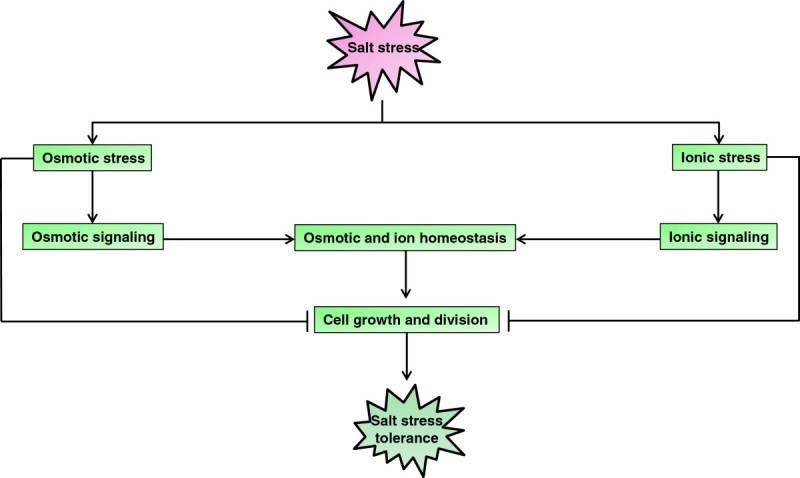
**Diagrammatic representation of salt stress response in rice.** Growth and division of the cell under salt stress depends on osmotic and ionic signaling.

**Figure 2:**
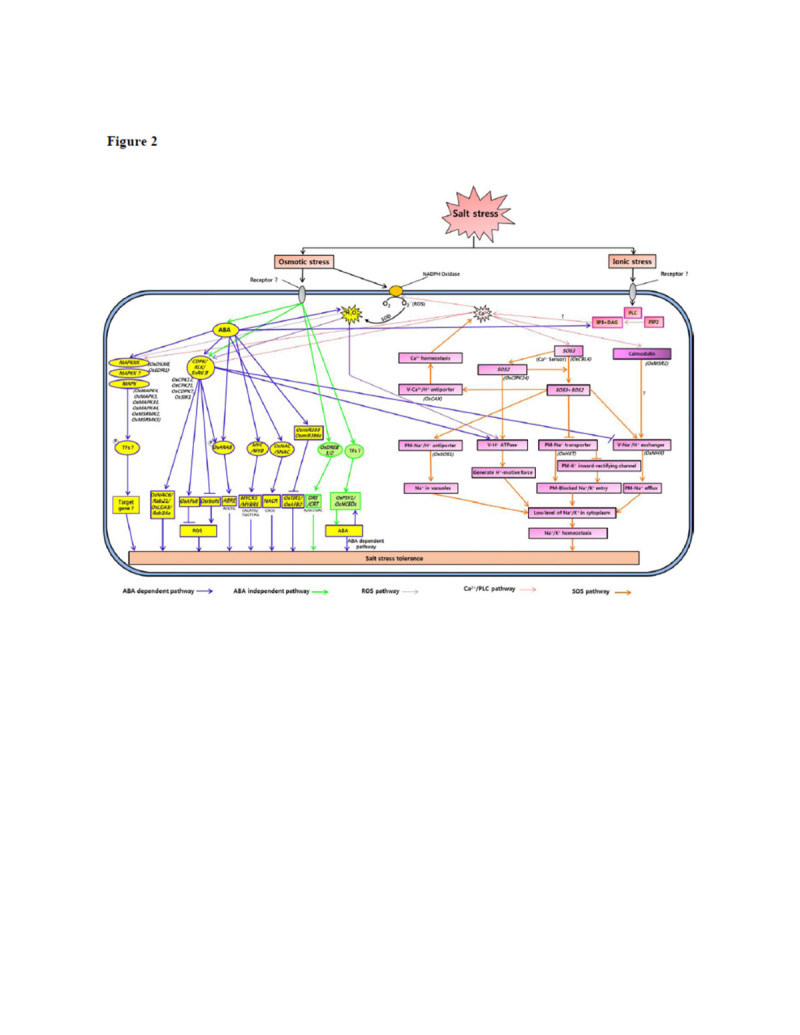
**Overall signaling pathway in rice during salt stress.** Salt stress evokes both osmotic and ionic stress. Osmotic stress signaling is transduced via ABA-dependent or ABA-independent pathway. ABA dependent pathway includes mitogen activated protein kinase (MAP Kinase) cascades, calcium-dependent protein kinases (CDPK), receptor-like kinases (RLK), SNF1-related protein kinases (SnRK), transcription factors (*OsRAB1*, *MYC/MYB* and *OsNAC/SNAC*) and micro RNAs. ABA-independent pathway includes transcription factors (*OsDREB1* and *OsDREB2*) and stress related genes (*OsPSY1*, *OsNCEDs*). Ionic stress does signaling via Ca^2+^/PLC pathway and salt overly sensitive (SOS) pathway and Calmodulin (CaM) pathway. Ca^2+^ is sensed by Ca^2+^ sensor (OsCBL4) and the sensor activates calcineurin B-like protein kinase (OsCIPK24), which in turns activates Na^+^/H^+^ antiporter (OsSOS1), H^+^/Ca^+^ antiporter (OsCAX1), vacuolar H^+^/ATPase, vacuolar Na^+^/H^+^ exchangers (OsNHX1) and suppress K^+^/Na^+^ symporter (OsHKT1) to maintain ionic homeostasis under salt stress. Ca^2+^ also activates calmodulin (OsMSR2) which also activates vacuolar Na^+^/H^+^ exchanger (OsNHX1). Blue arrow indicates ABA dependent pathway, green arrow shows ABA-independent pathway, violet arrow shows ROS pathway, red arrow shows Ca^2+^/PLC pathway and orange arrow shows SOS pathway.

Salt stress evokes both osmotic stress and ionic stress which inhibits the plant’s normal cell growth and division. To encounter the adverse environment, plants maintain osmotic and ion homeostasis with rapid osmotic and ionic signaling (Figure 1). Osmotic stress due to high salt rapidly increases abscisic acid (ABA) biosynthesis, thus regulating ABA-dependent stress response pathway. There are several salt stress inducible genes which are ABA-independent (Figure 2).

### Salt stress tolerance via ABA-dependent pathway

High salinity-induced osmotic stress increases the biosynthesis of ABA. ABA biosynthesis via terpenoid pathway starting from isopentenyl pyrophosphate (IPP) has been reviewed in rice (Ye et al. 2012). Among many genes involved in this pathway, a phytoene synthase gene, *OsPSY3* and 9-cis-epoxycarotenoid dioxygenases genes *(OsNCED3*, *OsNCED4* and *OsNCED5)* are induced one hour after salt stress and their expression is well correlated to the level of ABA in rice roots (Welsch et al. 2008). ABA then acts as a regulator initiating second round of signaling salt stress response in ABA-dependent pathway. Here we reviewed genes for protein kinases (Receptor-like kinases, *RLKs*; Mitogen activated protein kinases, *MAPKs*; SNF1-related protein kinases, *SnRKs*; Ca^2+^ dependent protein kinases, *CDPKs*), transcription factors (TFs), micro RNAs and reactive oxygen species (ROS) involved in the salt stress tolerance through ABA-dependent pathway (Figure 2).

In plant, protein kinases play important roles in regulating the stress signal transduction pathways. Receptor-like kinases (RLKs) have important roles in plant growth, development and stress responses. Salt, drought, H_2_O_2_ and ABA treatments induced the expression of a putative RLK gene, *OsSIK1*. Transgenic rice plants overexpressing *OsSIK1* (*OsSIK1*-*ox*) show higher tolerance to salt and drought stresses than control plants and the knock-out mutants *sik1* as well as RNA interference (RNAi) plants (Ouyang et al. 2010). Mitogen activated protein (MAP) kinase cascades play a crucial role in salt stress response in rice as well. Until now, at least two salt inducible MEKKs have been reported in rice. One of them is *OsEDR1* which is upregulated by various environmental stresses such as high salt, physical cutting and hydrogen peroxide (Kim et al. 2003). A putative MEKK mutant, *dsm1,* showed sensitivity to salt stress as well as drought stress than wild type plants (Ning et al. 2010). Although these genes are responsive to salt stress, there is no evidence that the MEKKs are regulating any downstream MKK. Several salt-inducible MAPKs have been reported in rice. Transcriptional regulation of *OsMAPK4* by salt, cold and sugar starvation was reported although its ABA-dependency is not clear (Fu et al. 2002). Biotic and abiotic stress inducible *OsMAPK5* has been cloned and overexpressed in rice which subsequently exhibited increased tolerance to salt, drought and cold stresses with increased kinase activity (Xiong et al. 2003). Expression of two novel MAPKs, *OsMSRMK2* and *OsMSRMK3* were induced by various environmental stresses suggesting their possible involvement in defense/stress response pathways (Agrawal et al. 2002,2003). A putative salt and ABA-inducible MAPK gene was introduced into transgenic rice and the plants exhibited higher Na^+^/K^+^ ratio than *OsMAPK44* suppressed plants under salt stress indicating that *OsMAPK44* may have a role in ion balance during salt stressed condition (Jeong et al. 2006). Overexpression of a drought stress inducible *OsMAPK33* in transgenic rice also revealed a result similar to that of *OsMAPK44* showing higher Na^+^/K^+^ ratio than wild type plants indicating the negative role of *OsMAPK33* in salt stress tolerance through unfavorable ion homeostasis (Lee et al. 2011). Although several salt stress-related MAPKs have been reported, the linkage to upstream MKK or to downstream targets are not clear yet (Singh and Jwa, 2013).

An ABA-dependent Ca^2+^-dependent protein kinases (CDPKs), *OsCPK21,* have been cloned and the *OsCPK21-ox* transgenic rice exhibited higher salt stress tolerance than wild-type plant with enhanced expression of the ABA and salt-stress inducible genes such as *OsNAC6* and *Rab21* (Asano et al. 2011). An SNF1-related protein kinase (SnRK) functions in salt stress tolerance as well. In rice, ten members of SnRK2 family have been shown to be activated by hyperosmotic stress through phosphorylation (Kobayashi et al. 2004). Among them *SAPK4* seems to play a role in the salt stress tolerance. *SAPK4*-*ox* transgenic rice revealed an improved salt tolerance with a reduced Na^+^ accumulation in the cytosol. The vacuolar Na^+^/H^+^ antiporter gene, *OsNHX1* is less expressed in the transgenic plants, indicating the reduced Na^+^ accumulation due to cellular Na^+^ exclusion rather than vacuolar sequestration of the ion (Diedhiou et al. 2008).

Transcriptional regulatory network of ABA-dependent TFs (Figure 2) is recently reviewed (Todaka et al., 2012). Promoter regions of ABA-inducible genes have conserved *cis*-acting element, ABRE, where bZIP-type TFs bind (Yamaguchi-Shinozaki and Shinozaki 2006; Todaka et al. 2012). A T-DNA insertion mutant of salt stress inducible bZIP TF, *OsABF2,* increased sensitivity to salt stresses compared to control plant indicating that *OsABF2* is a positive regulator of salt stress (Hossain et al. 2010). *OsABF2* binds to ABRE and its N-terminal region transactivated a downstream reporter gene in yeast. Overexpression of ABA-dependent stress inducible *OsbZIP23* enhanced tolerance to salt and drought stresses (Xiang et al. 2008). On the other hand*,* overexpression of salt and ABA responsive *OsABI5,* another bZIP-type TF gene showed high sensitivity to salt stress, whereas transgenic rice plants expressing antisense *OsABI5* showed increased salt stress tolerance. The OsABI5 protein is localized in the nucleus and binds to a G-box element (Zou et al. 2008). These opposing functions displayed by bZIP TFs, are still unclear. Microarray analysis combined with expressed sequence tag analysis of rice 89 *OsbZIP* genes revealed that no single gene was activated by only salt stress although several genes were activated by drought stress together (Nijhawan et al. 2008). This result may indicate that stress inducible OsbZIPs are mostly related to osmotic stress signaling via ABA.

MYB TFs containing a highly conserved DNA-binding MYB domain of 52 amino acids are also involved in the regulation of salt stress response via ABA-dependent pathway. Overexpression of a stress inducible *OsMYB3R-2* improved salt stress tolerance along with cold and drought stress tolerance in *Arabidopsis*, with increased expression of *DREB2A*, *COR15a* and *RCI2A* (Dai et al. 2007). Transgenic rice overexpressing *OsMYB2* also showed an enhanced salinity stress tolerance along with drought and cold stress tolerance without compromising the growth rate as compared with control (Yang et al. 2012). In the transgenic rice some putative downstream genes such as *OsLEA3*, *OsRab16A* and *OsDREB2A* were up regulated suggesting that *OsMYB2* encodes a stress-responsive MYB TF that may act as a master switch in the stress tolerance.

NAC-type TFs also regulate some salt-responsive genes through ABA-dependent manner. They were isolated initially from *Arabidopsis* by yeast one hybrid screening as TFs that regulate expression of a salt-inducible *ERD1* (Tran et al. 2004). High salinity stress induces several NAC genes in rice as well. Overexpression of salt stress inducible *SNAC1* (Hu et al. 2006) and *OsNAC6* showed improved tolerance to high salinity stress although growth retardation and low yield were exhibited in *OsNAC6-ox* under the non-stress condition (Nakashima et al. 2007). Recently, it was shown that *OsHDAC1* encoding a histone deacetylase epigenetically represses *OsNAC6* expression, so the root growth retardation of *OsNAC6* overexpressor is similar to the phenotype of *OsHDAC1* knock-out (Chung et al. 2009). OsNAC5, another salt inducible NAC TF, binds to the NAC recognition core sequence (CACG) of *OsLEA3* promoter and the transgenic overexpressor of *OsNAC5* also showed improved salt tolerance (Takasaki et al. 2010). The *OsNAC5* overexpression also correlated positively with accumulation of compatible solutes such as proline and soluble sugars (Song et al. 2011).

Zinc finger TFs were first recognized in *Xenopus* TFIIIA as a repeated zinc-binding motif containing conserved cysteine and histidine ligands (Miller et al. 1985). From rice, salt stress-responsive *OSISAP1-ox* conferred tolerance to salt, cold and dehydration stress in transgenic tobacco (Mukhopadhyay et al. 2004). TFIIIA-type *ZFP252–ox* rice also showed enhanced salt and drought tolerance with the elevated level of stress defense genes, as compared with *ZFP252* antisense and non-transgenic plants (Xu et al. 2008). Huang et al. (2009) have isolated *DST* (drought and salt tolerance) gene, which negatively regulates stomatal closure and directs modulation of genes related to H_2_O_2_ homeostasis. *DST* mutant increases stomatal closure and reduces stomatal density, thus resulting in enhanced salt and drought tolerance in rice. Another salt responsive zinc finger protein gene *ZFP179-ox* leads to increased salt stress tolerance with increased level of free proline and soluble sugars in transgenic rice (Sun et al. 2010). An increased level of expression of a number of stress-related genes, including *OsDREB2A*, *OsP5CS*, *OsProT*, and *OsLEA3* was observed in the transgenic rice. Recently, a salt inducible OsTZF1, a CCCH-type zinc finger protein, has been shown to bind to U-rich regions in the 3' untranslated region of mRNAs (Jan et al. 2013) and overexpression of *OsTZF1* showed improved tolerance to salt and drought stresses, *OsTZF1* was implicated to play a role in RNA metabolism of stress-responsive genes.

WRKY TFs are also involved in stress response. Overexpression of *OsWRKY13* reduced salt stress tolerance via antagonistic inhibition of *SNAC1* in rice indicating that *OsWRKY13* is a negative regulator of salt stress response (Qiu et al. 2008). Similarly, *OsWRKY45-2* suppressing lines showed increased salt stress tolerance with reduced ABA sensitivity (Tao et al. 2011). A salt-inducible AP2/ERF type TF gene, *OsERF922–ox* rice shows decreased tolerance to salt stress with an increased Na^+^/K^+^ ratio in the shoots (Liu et al. 2012). Apart from TFs many other downstream genes are involved in salt tolerance in rice. Hoshida et al. (2000) examined the increased photorespiration effect on the salt tolerance by overexpressing chloroplastic glutamine synthetase (*GS2*) gene from rice. *GS2-ox* rice showed increased salt stress tolerance retaining more than 90% activity of photosystem II in comparison to complete loss of photosystem II in control plant. Overexpression of a salt stress inducible JAZ protein gene, *OsTIFY11a,* resulted in increased salt and dehydration stress tolerance in rice, yet the function of the protein is not clear (Ye et al. 2009). *OsSKIPa* (Ski-interacting protein) expression is induced by various abiotic stresses and phytohormone treatments. Overexpression of *OsSKIPa,* also exhibited significantly improved growth performance in the salt and drought resistance with increased transcript levels of many stress-related genes, such as *SNAC1* and rice homologs of *CBF2*, *PP2C* and *RD22* (Hou et al. 2009).

Recently, it has been known that microRNAs (miRNAs) play a key role in the regulation of gene expression at the post-transcriptional level. Under salt-stressed condition, expression of two microRNAs, *osa-MIR396c* and *osa-MIR393*, decreased in ABA-dependent manner and overexpression of both miRNAs resulted to a reduced salt stress tolerance, which is lower than the wild-type plants (Gao et al. 2010,2011). This suggests that these miRNAs are have some role in the salt stress tolerance, although the molecular mechanism is not clear yet.

In barley *HVA1* encodes a late embryogenesis abundant (LEA) protein, which is thought as a molecular chaperon. Expression of *HVA1* gene led to significantly increased salt and drought tolerance in transgenic rice (Xu et al. 1996). A rice LEA gene, *OsLEA3-2,* was also overexpressed in rice as well as in yeast. When tested to salt stress, these transgenic organisms showed enhanced growth performance supporting the idea that LEA proteins play important role in the protection of plants under stressed conditions (Duan and Cai 2012). OsLEA3-2 was shown to protect lactate dehydrogenase from aggregation on dessication *in vitro*.

### Salt stress tolerance via ABA-independent pathway

There are several salt stress inducible genes which are ABA-independent. These include genes for DREB1 and DREB2 TFs, some kinases, spingolipid biosynthesis enzymes, and ROS-producing/scavenging enzymes. CBF/DREB-type genes first identified from *Arabidopsis* encode AP2/ERF domains that bind to a cis-acting element, DRE/CRT with a core sequence A/GCCGAC (Yamaguchi-Shinozaki and Shinozaki 2005; Todaka et al. 2012). Rice genome contains at least fourteen DREB-type genes, among which *OsDREB1A*, *OsDREB1F* and *OsDREB2A* are induced by salt stress and their overexpression displayed strong abiotic stress tolerance. Transgenic *Arabidopsis* or rice overexpressing *OsDREB1A*, *OsDREB1F* and *OsDREB2A* showed improved salinity tolerance (Dubouzet et al. 2003; Wang et al. 2008; Mallikarjuna et al. 2011). Microarray analysis revealed the target stress-inducible genes of *OsDREB1A* and *OsDREB2A* encoded proteins thought to function in stress tolerance in the plants, which is similar with the target genes of DREB1 and DREB2 proteins in *Arabidopsis* (Sakuma et al. 2006 Jeon and Kim 2013). DRE-containing promoter region of *OsDhn1* is activated by OsDREB1A and OsDREB1D (Lee et al. 2013). These observations showed that the DREB/CBF TFs are conserved in rice and *Arabidopsis* and DREB-type genes are useful for improvement of salt stress tolerance in transgenic rice.

ABA-independent kinases are also involved in salt stress tolerance. Overexpression of a CDPK, OsCDPK7 enhanced salt stress tolerance in transgenic rice and the extent of tolerance correlated well with the level of *OsCDPK7* expression (Saijo et al. 2000). It was found that knockout plants of *OsGSK1,* a negative regulator gene of brassinosteroid signaling, showed enhanced tolerance to salt as well as other stresses suggesting that BR plays important role for stress tolerance (Koh et al. 2007). *OsCPK12*, a member of CDPK family, negatively regulates the expression of *OsrbohI* while it positively regulates ROS detoxification by controlling the expression of *OsAPX 2* and *OsAPX 8* under high salinity condition. The accumulation of H_2_O_2_ in *OsCPK12*-ox plants during salt stress was less than that in WT plants, whereas in *oscpk12* and *OsCPK12* RNAi plants the accumulation was more (Asano et al. 2012). This shows that *OsCPK12* confers salt stress tolerance by repressing ROS accumulation rather than by affecting ABA mediated salt stress signaling.

ABA-independent ROS scavenging system is also involved in salt stress tolerance. A salt responsive malic enzyme gene in rice, *NADP-ME*, was ectopically expressed in *Arabidopsis* which resulted in enhanced salt stress tolerance probably due to the increased reducing power of ROS (Liu et al. 2007). A mitochondrial superoxide dismutase gene *Mn-SOD* from *Saccharomyces cerevisiae* was expressed in rice chloroplast. The transgenic rice plants failed to show salt tolerance but decrease of SOD activities was slower than those of wild type at high salinity condition (Tanaka et al. 1999). Recently, *katE* encoding catalase from *E. coli* was transformed into rice which subsequently showed higher salt stress tolerance than the wild type with enhanced level (1.5 to 2.5 fold) of the catalase activity. Alternative oxidase (AOX) is an inner mitochondrial membrane protein that functions as terminal oxidase in the alternative (cyanide resistant) pathway, and AOX serves as oxidation stress reliever from environmental stresses, particularly salt and dehydration stress (Purvis 1997; Cournac et al. 2002). An AOX gene in rice OsIM1 was identified as salt responsive gene by using differential display method indicating the role of AOX pathway under salt stress (Kong et al. 2003). This implicates the importance of ROS scavenging system in the plant salt stress tolerance (Motohashi et al. 2010). Salt stress supportive genes described in this section is reported in supplemental information (Additional file 1: Table S1).

### Signaling molecules regulating salt stress in rice

Under salinity stress conditions, diverse signaling molecules such as phospholipids, hormones and calcium ions (Ca^2+^) regulate stress signaling pathways for maintaining an osmotic adjustment or homeostasis and regulating plant growth and development. Plant hormonal regulations and Ca^2+^ dependent modification of enzymatic activities are coordinately or independently integrated into the stress signaling pathways. In this section, we reviewed the recent advances in molecular mechanisms of how these signaling molecules are concerted to cytosolic and nuclear events for maintaining ionic homeostasis and salt stress tolerance in plants.

### Phospholipids and Ca^2+^ ions

As signaling molecules, phospholipids including IP3 (Inositol triphosphate) DAG (Diacylglycerol) and PA (phosphatidic acid) seem to play an important structural roles during stress responses in inducing cytosolic Ca^2+^ spiking. Although the precise roles of phospholipid-based signaling in plants are still underexplored, some recent evidences have revealed their possible involvement in salt stress tolerance (Zhu 2002). Under stress conditions, PA and IP3 levels were rapidly increased in rice, *Arabidopsis* and tobacco (Zhu 2002; Darwish et al. 2009). Furthermore, several studies have shown that IP3 and its biosynthetic related genes rapidly increased in response to hyperosmotic stress and stress hormone ABA treatment (Drobak and Watkins 2000; DeWald et al. 2001). The formation of the phospholipid-based signaling molecules is mainly regulated by phospholipase C and D (PLC/ PLD). IP3 act as strong elicitors in mobilizing cytosolic Ca^2+^ levels in plants (Zhu 2002). This implies the activation of phospholipid formations for salt stress-induced cytosolic Ca^2+^ spiking possibly by membrane anchored salt signaling sensor proteins.

It has been well established that high salt stress rapidly leads to cytosolic Ca^2+^ spiking. This event spontaneously initiates the stress signaling pathways for stress tolerance via stimulating various Ca^2+^ binding proteins including CBL-CIPKs, CDPKs and calmodulins (Mahajan et al. 2008; Kader and Lindberg 2010). Direct evidences of the essential role of Ca^2+^ spiking in salt tolerance are provided by identification of *Arabidopsis sos3* (*Salt Overlay Sensitive3*) mutants which are oversensitive to salt stress (Mahajan et al. 2008). The *SOS3* encodes an EF-hand type calcineurin B-like protein (CBL) and functioned in sensing the cytosolic Ca^2+^ concentration by direct binding to Ca^2+^. Indeed, the loss-of-function *sos3-1* mutation reduced its Ca^2+^ binding capacity, indicating that Ca^2+^ sensing by SOS3 is an essential mechanism for salt tolerance in plant (Sanchez-Barrena et al. 2004,2005). The Ca^2+^ bound CBL proteins directly activate their interacting CIPK (CBL-interacting protein kinase) proteins. As a SOS3 interacting CIPK, SOS2 (*Salt Overlay Sensitive2*) was identified, and the activation of kinase activity of SOS2 by SOS3 was in a Ca^2+^ dependent manner (Halfter et al. 2000; Liu et al. 2000). Also, SOS2 interacts and activates vacuolar N^+^/H^+^ and H^+^/Ca^2+^ antiporters and V-ATPase independently of SOS3 leading to sequestration of excess Na^+^ ion into vacuoles and maintain cytosolic Ca^2+^ level (Qiu et al. 2004; Ji et al. 2013). SOS pathway is conserved in rice and a Na^+^/H^+^ antiporter, OsSOS1, was shown that OsSOS1 in the plasma membrane of yeast (*Saccharomyces cerevisiae*) cells reduced total Na^+^ content in the cell. Other SOS2 and SOS3 homologs in rice were also identified as OsCIPK24 and OsCBL4, respectively (Atienza et al. 2007). Xiang et al. (2007) surveyed 30 putaitive CIPK genes from rice genome and found many of them showed stress responsive expression. Among those, they showed that OsCIPK15-ox transgenic rice have significantly improved tolerance to salt stress. These suggest that Ca^2+^ spiking by salt stress triggers the SOS3-SOS2 mediated salt stress signaling pathways. Further, these demonstrate that the high degree of functional conservation of Ca^2+^-mediated sodium ion homeostasis is well evolved in monocot and dicot plants. More detailed studies on the precise roles of rice SOS pathways will be necessary to extend our understanding in the molecular mechanisms of maintaining an osmotic adjustment or homeostasis. These efforts will be very helpful for developing biotechnological tools to increase osmotic and salt tolerances of crop plants.

### Abscisic acid (ABA)

Plant stress hormone ABA has long been considered as an essential phytohormone for regulating various plant developmental processes as well as the adaptive responses to broad range of abiotic stresses (Zhu 2002 Hadiarto and Tran 2011). Indeed, the endogenous level of ABA and its biosynthetic genes in plant is rapidly increased by abiotic stresses including drought and salt stress. The elevated ABA hormone aids plant to acclimate under lower water availability by closing guard cells and accumulating numerous proteins for osmotic adjustment. Interestingly, the expression of many ABA biosynthetic genes seems to be regulated by a stress-induced Ca^2+^-dependent phosphorylation and its signaling pathways in rice (Du et al. 2010; Saeng-ngam et al. 2012). For example, overexpression of drought-responsive *OsDSM2 (Drought-hypersensitive mutant2)* and *OsCam1-1* genes led to accumulation of ABA and tolerance to salt stress in rice. *OsDSM2* and *OsCam1-1* genes encode an ABA biosynthetic β-carotene hydrolase and a Ca^2+^-binding calmodulin, respectively (Du et al. 2010; Saeng-ngam et al. 2012). These results suggest that stress-activated Ca^2+^ spiking could provide the positive feedback loop for ABA biosynthesis, and this event might be critical for stress tolerance in rice.

### Jasmonate (JA)

JA including Methyl Jasmonate (MeJA) and its free-acid form, JA, is an important signaling molecule for diverse developmental processes and defense responses (Kazan and Manners 2012). Several studies have investigated biological relevancies of JA signaling in salt stress in rice. Interestingly, higher endogenous JA contents were observed in salt-tolerant cultivar rice than in salt-sensitive cultivar (Kang et al. 2005). In addition, MeJA level was increased by high salt stress in rice (Moons et al. 1997), supposing that high accumulation of JA in rice could be an effective protection against salt stress. Consistently, exogenous JA treatment dramatically reduced the Na^+^ ions in salt-tolerant cultivar rice (Kang et al. 2005).

Recent findings showed some evidences of crosstalk between ABA and Jasmonate (JA) in regulating salt stress (Shinozaki and Yamaguchi-Shinozaki 2007). JA is involved in development and plant defense responses through modulating JAZ (Jasmonate ZIM-domain) transcription factors. Interestingly, MYC2 transcription factors are commonly used for regulating gene expression of JA, ABA and salt stress. JA induces proteolysis of JAZ which functions in direct repressing MYC2, thereby enabling MYC2 transcription factors to activate the downstream target gene expressions (Kazan and Manners 2012). This suggests that JA plays important roles in ABA-dependent regulation of salt stress responsive genes. Several rice JAZ proteins such as *OsTIFY1, 6, 9, 10* and *11* have been identified as salt-inducible genes (Ye et al. 2009). However, the antagonistic roles of JA in ABA-mediated regulations of salt-stress related gene expressions have been also reported in rice root. JA treatment effectively reduced the ABA-mediated up-regulation of *OsLEAs* in rice root. Furthermore, JA-inducible genes were not stimulated in the presence of JA and ABA (Moons et al. 1997). Taken together, this implies the involvement of different regulation mechanisms in JA and ABA-mediated responses to salt stress.

### Brassinosteroids (BRs)

Plant steroid hormone BRs play essential roles in diverse plant developmental processes and stress tolerances (Vriet et al. 2012). Recently, many studies have demonstrated the positive roles of BR applications or endogenous BR contents in salt and drought stress in plant (Koh et al. 2007; Manavalan et al. 2012). BRs positively regulate salt stress responses in rice. A T-DNA inserted loss-of-function rice *gsk1* mutant, an orthologous gene of a BR negative regulator, BIN2, showed an increased tolerance to salt stress compared to wild type rice (Koh et al. 2007). Furthermore, exogenous BR application could remove the salinity-induced inhibition of seed germination and seedling growth in rice. Consistently, increasing endogenous sterol contents in rice by RNAi-mediated disruption of the rice *SQS* (*Squalene synthase*) gene led to decreasing stomata density and increasing drought stress tolerance (Manavalan et al. 2012). These results describe the role of BRs in tolerance to salt and drought stress. Nonetheless, the molecular mechanisms for BR-mediated salt stress tolerance are still unclear. Some possible molecular mechanisms linked BRs with salt stress acclimation were recently demonstrated. BRs might enable plant to resist salt stress condition by reducing stomatal conductance and ER stress signaling (Che et al. 2010; Kim et al. 2012). BR signaling pathways reduced the stomata density by suppressing the BIN2 triggered inactivation of YDA-mediated stomata development signaling cascades (Kim et al. 2012), indicating that higher BR activity could decrease water loss under drought and salt stress conditions. Another possibility of BR-mediated salt stress tolerance was reported (Che et al. 2010). RIP (Regulated intermembrane proteolysis) of miss-folding proteins occurred in ER by diverse stress conditions is well conserved mechanisms in eukaryotes. Two RIP related bZIP transcription factors are tightly linked with BR signaling pathways and this link is required for acclimation to numerous stresses (Che et al. 2010), suggesting the prominent roles of BRs in salt stress tolerances via ER stress signaling pathways.

### Genomics of osmoprotectants

Severe osmotic stresses, salinity, drought, and cold, cause detrimental changes in cellular components. Accumulation of certain organic solutes (known as osmoprotectants) is a common metabolic adaptation found in diverse plant species. The osmoprotectants have been definitely proven to be among the most important factors to protect plant cells from dehydration and salinity (Rontein et al. 2002; Yamaguchi-Shinozaki 2002). The organic solutes protect plants from abiotic stress by osmotic adjustment, detoxification of reactive oxygen species (ROS) and stabilization of the quaternary structure of proteins (Bohnert and Jensen 1996). Transgenic plants overexpressing the genes participating in the synthesis or accumulation of osmoprotectants that function for osmotic adjustment, such as proline (Kishor et al. 1995), glycinebetaine (Holmström et al. 2000) or other osmolytes show increased salt tolerance. The most important plant osmoprotectants are proline, glycine betaine, trehalose and myo-inositol. An important feature of osmoprotectants is that their beneficial effects are generally not species-specific, so that alien osmoprotectants can be engineered into plants and protect their new host.

### Proline

Proline as an amino acid is essential for primary metabolism in plants during salt and drought stresses, showing a molecular chaperone role due to its stabilizing action either as a buffer to maintain the pH of the cytosolic redox status of the cell (Verbruggen and Hermans 2008; Kido et al. 2013) or as antioxidant through its involvement in the scavenging of free highly reactive radicals (Smirnoff and Cumbes 1989) or acting as a singlet oxygen quencher (Bhalu and Mohanty 2002). In higher plants, proline biosynthesis may proceed either via glutamate, by successive reductions catalyzed by pyrroline-5-carboxylate synthase (P5CS) and pyrroline-5-carboxylate reductase (P5CR) or by ornithine pathway and ornithine d-aminotransferase (OAT), representing generally the first activated osmoprotectant after stress perception (Savouré 1995; Parida et al. 2008).

Proline accumulation in transgenic rice plants with *P5CS* cDNA was reported and proved stress-induced overproduction of the P5CS enzyme under salinity stress (Zhu et al. 1998; Lee et al. 2012). A cDNA clone encoding *P5CS* was later isolated from rice and characterized. The expression of *P5CS* and the accumulation of proline in salt tolerant cultivar are much higher than in salt sensitive lines (Igarashi et al. 1997). When *P5CS* gene was overexpressed in the transgenic tobacco plants, an increased production of proline coupled with salinity tolerance were noted (Kishor et al. 1995). Thus, *P5CS* may not be the rate-limiting step in proline accumulation (Delauney and Verma 1993).

### Glycine betaine

Betaines are amino acid derivatives in which the nitrogen atom is fully methylated such as that of quaternary ammonium compounds. Among the many quaternary ammonium compounds known in plants, glycine betaine (GB) occurs most abundantly in response to dehydration stress (Yang et al. 2003) where it reduces lipid peroxidation, thereby helps in maintaining the osmotic status of the cell to ameliorate the abiotic stress effect (Chinnusamy et al. 2005). In higher plants, glycine betaine is synthesized in the chloroplast from serine via choline by the action of choline monooxygenase (CMO) and betaine aldehyde dehydrogenase (BADH) enzymes (Ashraf and Foolad 2007).

Genes involved in osmoprotectant biosynthesis are upregulated under salt stress, and the concentrations of accumulated osmoprotectants correlates with osmotic stress tolerance (Chen and Murata 2002; Zhu 2002). Choline dehydrogenase gene (*codA*) from *Arthrobacter globiformis* aids in improving the salinity tolerance in rice (Vinocur and Altman 2005).

Tolerant genotypes normally accumulate more glycine betaine than sensitive genotypes in response to stress. This relationship, however, is not universal. The osmolyte that plays a major role in osmotic adjustment is species dependent. Some plant species such as rice (*Oryza sativa*), mustard (*Brassica* spp.), *Arabidopsis* (*Arabidopsis thaliana*) and tobacco (*Nicotiana tabacum*) naturally do not produce glycine betaine under stress or non-stress conditions (Rhodes and Hanson 1993). In these species, transgenic plants with overexpressing glycine betaine synthesizing genes exhibited abundant production of glycine betaine, which leads plants to tolerate stresses, including salinity stress (Rhodes and Hanson 1993). The limitation in production of glycine betaine in high quantities in transgenic plants is reportedly due to either low availability of substrate choline or reduced transport of choline into the chloroplast where glycine betaine is naturally synthesized (Huang et al. 2000). Thus, to engineer plants for overproduction of osmolytes such as glycine betaine , other factors such as substrate availability and metabolic flux must also be considered.

In rice indica plant (cv. IR36), deficiency of glycine betaine has been attributed to the absence of the two enzymes, choline monooxygenase and betaine aldehyde, in the biosynthetic pathway (Rathinasabapathi et al. 1993). However, the enzymatically active BADH is detectable in Japonica variety of rice (cv. Nipponbare) (Nakamura et al. 1997). This apparent discrepancy is yet subject for further investigation. Exogenous foliar application of glycine betaine to *Oryza sativa* (Harinasut et al. 1996) resulted in improved growth of plants under salinity stress condition. Further, a decrease in Na^+^ and an increase in K^+^ concentrations in shoots were observed in GB-treated plants under salinity. This indicates the possible role of glycine betaine in signal transduction and ion homeostasis as well.

### Trehalose

Trehalose is a non-reducing disaccharide in which two glucose molecules are joined together by a glycosidic -(1–1) bond. In plants, the synthesis of this sugar occurs normally by the formation of the trehalose-6-phosphate (T6P) from the UDP-glucose and glucose-6-phosphate, a reaction catalyzed by the trehalose 6-phosphate synthase (TPS). Afterwards the T6P is dephosphorylated by the trehalose-6-phosphate phosphatase (TPP) resulting in the formation of free trehalose (Wingler 2002). It has been shown that trehalose can protect proteins and cellular membranes from denaturation caused by a variety of stress conditions, including desiccation (Elbein et al. 2003).

Trehalose overproduction has considerable potential for improving abiotic stress tolerance in rice transgenic plants. Increased trehalose accumulation showed higher level of tolerance to salt, drought, and low-temperature stresses, as compared with the nontransformed controls (Garg et al. 2002). Trehalose may ameliorate salinity stress through stabilization of the plasma membranes, since it decreased the rate of ion leakage and the rate of lipid peroxidation of the root cells, and increased the ratio of K^+^/Na^+^ ions in the leaves of maize seedlings (Zeid 2009).

### Myo-inositol

Inositol is a cyclohexanehexol, a cyclic carbohydrate with six hydroxyl groups, one on each carbon ring. Among the nine types of existing steroisomers, myo-inositol is the most abundant in the nature, being also important for the biosynthesis of a wide variety of compounds including inositol phosphates, glycosylphosphatidylinositols, phosphatidylinositides, inositol esters, and ethers in plants (Murthy 2006). Myoinositol serves as a substrate for the formation of galactinol, the galactosyl-donor that plays a key role in the formation of raffinose family oligosaccharides (RFOs, raffinose, stachyose, verbascose) from sucrose. RFOs accumulate in plants under different stress conditions (Peters et al. 2007). In the case of the halophyte *Mesembryanthemum crystallinum* (common ice plant) - that possesses a remarkable tolerance against drought, high salinity, and cold stress inositol is methylated to D-ononitol and subsequently epimerized to D-pinitol. This plant accumulates a large amount of these inositol derivatives during the stress (Vernon et al. 1993). Due to the potential of myo-inositol, some transgenic plants expressing this substance have been generated, mainly using MIPS enzyme or inositol derived enzymes. Isolation of the *PINO1* gene (also known as *PcINO1*, encoding an l-myo-inositol 1-phosphate synthase) from the wild halophytic rice relative *Porteresia coarctata* and transformation in tobacco has been reported (Majee et al. 2004). This gene conferred tobacco plants tolerance to 200–300 mM NaCl keeping up 40–80% of the photosynthetic competence with concomitant increased inositol production, which is significantly better than the unstressed control. Additionally, *PINO1* transgenics showed *in vitro* salt-tolerance, complementing *in planta* functional expression of this gene.

### Genomic overview of transporters in rice salt response

**Figure 3:**
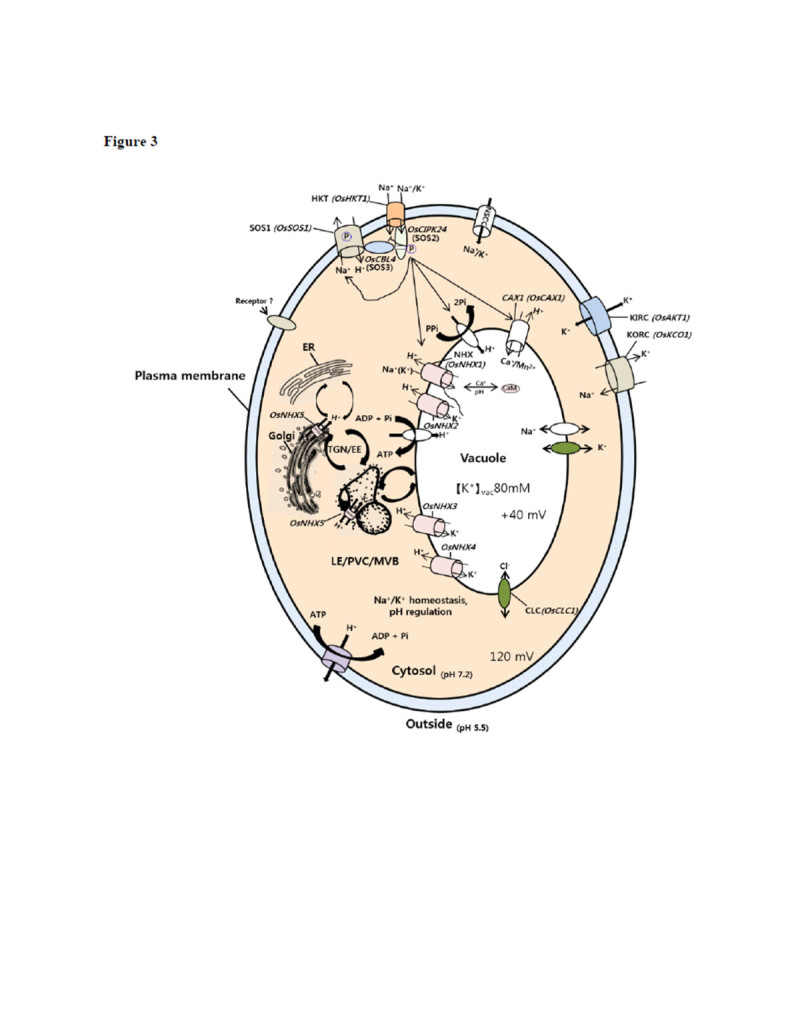
**Schematic diagram of a plant cell showing regulation of ion homeostasis by various ion transporters.** The salinity stress signal is perceived by receptor(s) or salt sensor(s) probably present at the plasma membrane of the cell. The signal is responsible for activating the SOS pathway, the component of which helps in regulating some of these transporters. The transporters are K^+^ inward-rectifying channel (OsAKT1), K^+^/Na^+^ symporter (OsHKT1), nonselective cation channel (NCC), K^+^ outward-rectifying channel (OsKCO1), Na^+^/H^+^ antiporters (OsSOS1), vacuolar Na^+^/H^+^ exchangers (OsNHX1-4), endosomal Na^+^/H^+^ exchanger (OsNHX5), H^+^/Ca^+^ antiporter (OsCAX1) and vacuolar chloride channel (OsCLC1). Na^+^ extrusion from plant cells is powered by the electrochemical gradient generated by H^+^-ATPases, which permits the Na^+^/H^+^ antiporters to couple the passive movement of H^+^ inside along the electrochemical gradient and extrusion of Na^+^ out of cytosol. ER, endoplasmic reticulum; TGN/EE, trans-Golgi network/early endosome; LE/PVC/MVB, late endosome/pre-vacuolar compartment/multivesicular body; RE, recycling endosome. The stress signal sensed by SOS3 activates SOS2, which activates SOS1, details is given in the text.

Various channels, carriers and pumps are working for the ion homeostasis in the cell and salt stress causes ions unbalance (Figure 3). In rice, 1,200 transporter proteins have been annotated from the genome sequence, among which 84% are active transporters (Nagata et al. 2008). Here we briefly reviewed ion transporters involved in the salt tolerance.

### Na^+^/H^+^ antiporters

The importance of Na^+^ transporters for Na^+^ tolerance in plant was first known in sugar beets (Blumwald and Poole, 1987). Activity and function of Na^+^/H^+^ antiporters, major Na^+^ transporters in plants, have been studied in various plants including rice and *Arabidopsis*. In *Arabidopsis*, the plasma membrane Na^+^/H^+^ antiporter (SOS1) regulates sodium efflux in roots and the long-distance transport of sodium from roots to shoots (Wu et al. 1996). A functional homologue of *SOS1* in rice, *OsSOS1*, had been isolated and a T-DNA insertion mutant of *OsSOS1* exhibited a salt sensitivity. It has been reported that SOS1 proteins contain self-inhibitory domains at their carboxy termini and the truncation of this inhibitory domain in OsSOS1 resulted in a much greater Na^+^ transport activity with enhanced salt tolerance in yeast cells (Atienza et al. 2007). In rice genome 13 antiporters are retrieved through *in silico* analysis, nine of which are Na^+^/H^+^ antiporters and 4 are K^+^/H^+^ antiporters, whereas 35 antiporters were retrieved in *Arabidopsis* with 29 being Na^+^/H^+^ antiporters and 6 K^+^/H^+^ antiporters.

In rice, 4 vacuolar Na^+^/H^+^ antiporters (*OsNHX1-4*) and one endosomal Na^+^/H^+^ antiporter (*OsNHX5)* have been reported (Bassil et al. 2012). OsNHX3 was showed to be phosphorylated at S471 in the C-terminus, a residue that is conserved in other vacuolar isoforms, suggesting that S471 is important for the activation of the antiporters. Overexpression of *OsNHX1* in rice and in maize improved salt tolerance by enhancing the compartmentalization of Na^+^ into the vacuoles (Chen et al. 2007). Ca^2+^/H^+^ antiporter (CAX) is a pump which helps intracellular Ca^2+^ ion homeostasis (Figure 3). From rice genome sequence four *CAX* genes (*OsCAX1a, OsCAX1b, OsCAX2 and OsCAX3*) and *OsCAX1c*, a pseudogene of *CAX1,* were retrived. Expression of all rice *CAX* genes except *OsCAX2* showed Ca^2+^ tolerance in yeast (Kamiya et al. 2004,2005). It is noticeable that in a salt tolerant rice cultivar Fl, the expression of a CAX-type exchanger was down-regulated compared to that of a salt sensitive cultivar RI, suggesting that the down-regulation of the CAX exchanger may be related to salt tolerance (Senadheera et al. 2009). However it is yet unknown whether or not CAX genes play direct role in the salt stress tolerance in rice.

### Na^+^/K^+^ symporter

HKT is a Na^+^/K^+^ symporter or Na^+^ uniporter present in the plasma membrane of plant cells. Rice genome has seven *HKT* transporter genes and two pseudogenes whereas *Arabidopsis* has 16 *HKT* transporter genes (Garciadeblas et al. 2003 Platten et al. 2006). Horie et al. (2007) reported that *oshkt2;1* mutant showed significant growth defect when a moderate amount of Na^+^ exists in K^+^- deficient condition. These results indicated that the OsHKT2;1-dependent Na^+^ influx in K^+^- deficient roots is regulated to prevent Na^+^ toxicity due to mass flow of Na^+^ through OsHKT2;1 (Figure 2.) A QTL, *SKC1*, from a salt tolerant variety which maintained K^+^ homeostasis under salt stress was mapped (Ren et al., 2005). Isolation of *SKC1* gene, which encodes HKT-type Na^+^-transporter suggested the role of *SKC1* in K^+^/Na^+^ homeostasis under salt stress.

H^+^-ATPase are primary active transporters. An electrochemical gradient generated by H^+^-ATPase helps in Na^+^ extrusion out of the cytosol. *In silico* analysis of rice genome revealed that 11 H^+^-ATPases are present in the vacuole, plasma membrane and Trans Golgi Network (TGN). In *Arabidopsis*, mutant lacking vacuolar V-H^+^-ATPase subunits showed a reduced tonoplast V-ATPase activity, but did not show sensitivity to high salinity (Krebs et al. 2010). However, a knockout of an endosomal (EE/TGN) *V-H*^*+*^*-ATPase* mutant showed increased salt sensitivity, indicating the importance of the endosomal system for the salt tolerance. In rice, expression of *H*^*+*^*-ATPase* gene, *OSA3,* under salt stress was greatly induced in a salt-tolerant mutant M-20, but not in a salt-sensitive variety 77–170, suggesting an active role of *OSA3* in relation with salt stress tolerance. However, there is no direct evidence that any of H^+^-ATPases in rice are involved in the salt stress tolerance.

### Channel protein

Several channel proteins are also involved in salt stress response in rice. Nonselective cation channels (NCCs) are proposed as an entry gate of Na^+^ into the plant cell. It was hypothesized that NCCs play a significant role in root Na^+^ uptake because of similarity between Na^+^ current and Ca^2+^ inhibition of radioactive Na^+^ influx (Demidchik et al. 2007). However, the exact conductance and proportion of this pathway may vary. Chloride ions are also important for salt stress response and chloride channel (CLC) although other channels are also involved in chloride transport. *In silico* analysis revealed that rice genome has 9 chloride channel (*CLC*) genes. Those channel proteins are present in vacuole, Golgi body and chloroplast. A salt stress inducible *OsCLC1* was identified from rice and the OsCLC1 was shown to operate as anion channels in one system, but H^+^/Cl^-^ antiporter in another (Nakamura et al. 2006). Although there is no direct evidence that *OsCLC* genes have roles in the salt stress tolerance, a comparison study revealed that there was a genotype-dependent differences in expression of *OsCLC1.* Under salt stress, salt-sensitive IR29 had repressed expression of *OsCLC1*, while salt-tolerant Pokkali showed induction particularly in roots, suggesting that the level of *OsCLC1* expression is correlated to the salt tolerance (Diedhiou and Golldack 2006). Inward-rectifying K^+^ channels (KIRC) mediates the influx of K^+^ on the plasma membrane and it selectively accumulates K^+^ over Na^+^ upon the plasma membrane hyperpolarization (Muller-Rober et al. 1995; Golldack et al. 2003). In rice genome, 15 are retrieved *in silico* compared to 12 in *Arabidopsis*. A few KIRC-encoding genes (*AKT*) have been functionally characterized. Salt stress inhibited *OsAKT1* gene expression and the inward K^+^ influx was significantly decreased in root protoplasts by salt stress, suggesting that OsAKT1 is a dominant salt-sensitive K^+^ uptake channel (Fuchs et al. 2005). Recently, it was shown that overexpression of a bHLH-type transcription factor gene *OrbHLH001* in transgenic rice increased the salt tolerance of transgenic rice with increased level of *OsAKT1* gene expression (Chen et al. 2013). More detailed studies on the precise roles of rice transporter genes will be helpful in understanding the molecular mechanisms of maintaining an osmotic adjustment or homeostasis and these efforts will be helpful for developing salt tolerant rice plants.

## Conclusion

Availability of high quality rice genome sequence fast tracked the progress made in functional genomics of salinity tolerance in rice. This aids in the discovery of several genes that could be deployed for use in breeding for rice salt tolerance. The complex mechanism underlying salt tolerance as well as the complex nature of salt stress itself and the wide range of plant responses make the trait unexplainable. Even so, several evidences showed that members of protein families involved in signal transduction, osmoregulation, ion transportation and protection from oxidative damage are critical in governing high salt tolerance (Figure 2). Various multiple signaling pathways can be activated during exposure to stress, leading to similar responses to different stimuli which suggest the overlap in gene expression between environmental stresses. Rice exhibits cellular ion homeostasis and enormous genetic variability in its sensitivity to salt stress. The indica varieties Pokkali and Nonabokra have higher endogenous ABA level during osmotic shock and are classified as highly salt tolerant ecotypes. To maximize the productivity of rice under saline soils there is an urgent need to look for sources of genetic variation that can be used for developing new cultivars with greater yield potential and stability over seasons and ecogeographic locations. Identification of molecular markers associated with salt stress tolerance genes or QTL conferring tolerance to high salinity has been demonstrated. Significant breakthroughs have been made on the mechanism and control of salinity stress tolerance in rice, but large gaps about our understanding in this field remained to be explored. Thus, further investigations are needed to sufficiently explain the underlying mechanisms of protection of rice under salt stress condition. Identification of the role of different component providing salt stress and the cross talks between these components will be a future challenge to disentangle the complete genome network of rice providing salinity tolerance. An emerging scope to identify novel cis-acting elements and elements acting in tandem may possibly lead to unraveling the complex web pattern for salinity signaling. Apparently, development of plants with improved tolerance to salt remained a big challenge despite the significant progress in genomics of salt tolerance in rice.

## Electronic supplementary material

Additional file 1: Table S1: List of salt stress supportive genes described in this paper. (PDF 134 KB)

Below are the links to the authors’ original submitted files for images.

Authors’ original file for figure 1

Authors’ original file for figure 2

Authors’ original file for figure 3
