# Supplementary material for: Insights into genomics of salt stress response in rice
Source: Rice (N Y). 2013 Oct 28;6:27. doi: 10.1186/1939-8433-6-27 (PMC4883734; doi:10.1186/1939-8433-6-27)
Supplement: Supplementary file 1 — Additional file 1: Table S1: List of salt stress supportive genes described in this paper. (PDF 134 KB) [file 12284_2013_61_MOESM1_ESM.pdf]

Addition File 1

Table S1. List of Salt Stress Supportive genes described in this paper

Gene name	Salt Stress Response	Source	Role in stress response	Reference
**Osmotic Stress**				
**Salt stress related gene in ABA dependent pathway**				
OsPSY3/ OsNCED3/ OsNCED4/ OsNCED5	Up-regulated	*Oryza sativa*	Regulate salt stress tolerance through ABA biosynthesis	Welsch et al. 2008
OsABF2	Down-regulated	*Oryza sativa*	Gene expressed modulation through an ABA-dependent pathway	Hossain et al. 2010
OsABI5	Up-regulated	*Oryza sativa*	Transgenic rice plants overexpressing *OsABI5* showed high sensitivity to salt stress	Zou et al. 2008
OsbZIP23	Up-regulated	*Oryza sativa*	Regulation of expression of genes involved in stress response and tolerance	Xiang et al. 2008
SAPK1/ SAPK2	Up-regulated	*Oryza sativa*	Regulation of expression of genes involved in stress response and tolerance	Kobayashi et al. 2004
OSRK1	Up-regulated	*Oryza sativa*	Regulation of expression of genes involved in stress response and tolerance	Kobayashi et al. 2004
SAPK4	Up-regulated	*Oryza sativa*	Maintain Na^+ and Cl^- homeostasis by reducing the *NHX* and *CLC1* expression	Diedhiou et al. 2008, Nakamura et al. 2006
OsMYB3R-2	Up-regulated	*Oryza sativa*	Regulation of stress responsive gene expression	Dai et al. 2007
OsMYB2	5hr (Up-regulated) 24hr (Down-regulated)	*Oryza sativa*	Overexpression of *OsMYB2* in rice seedlings functions as positive regulator to salt, cold, and dehydration stress tolerance.	Yang et al. 2012
OsLEA3/OsRab16A/OsDREB2A	Up-regulated	*Oryza sativa*	*OsMYB2* downstream genes	Yang et al. 2012
OsNAC6	Up-regulated	*Oryza sativa*	Regulation of stress responsive gene expression	Nakashima et al.2007
OsNAC5	Up-regulated	*Oryza sativa*	Regulation of OsLEA3 expression	Takasaki et al. 2010, Song et al. 2011
SNAC1	Up-regulated	*Oryza sativa*	Play important role in enhancing salt stress tolerance	Hu et al. 2006
ZFP252	Up-regulated	*Oryza sativa*	Accumulation of sugars and proline	Xu et al. 2008
OSISAP1	Up-regulated	*Oryza sativa*	Play important role in enhancing salt stress tolerance	Mukhopadhyay

				et al. 2004
DST	Down-regulated	*Oryza sativa*	Regulation H_2O_2-induced stomatal closure and negatively regulation	Huang et al. 2009
ZFP179	Up-regulated	*Oryza sativa*	Accumulation of sugars and proline	Sun et al. 2010
OsTZF1	Up-regulated	*Oryza sativa*	Play important role in enhancing salt and drought stress tolerance	Jan et al. 2013
OsCPK21	Up-regulated	*Oryza sativa*	Positive regulate the signaling pathways that are involved in the response to ABA and salt stress tolerance	Asano et al. 2011
OsNAC6/Rab21 /OsLEA3	Up-regulated	*Oryza sativa*	*OsCPK21* downstream genes	Asano et al. 2011, Duan et al. 2012
OsDCPK7	Up-regulated	*Oryza sativa*	Play important role in enhancing salt stress tolerance	Saijo et al. 2000
Rab16a /SalT /wsi18	Up-regulated	*Oryza sativa*	*OsCDPK7* downstream genes	Saijo et al. 2000
OsSIK1.	Up-regulated	*Oryza sativa*	*Play important role in enhancing salt stress tolerance*	Ouyang et al. 2010
OsDSM1	Up-regulated	*Oryza sativa*	Early signaling component in regulating responses to osmotic stress by regulating scavenging of ROS	Ning et al. 2010
OsEDR1	Up-regulated	*Oryza sativa*	An essential positive regulator of tolerance to salt stress.	Kim et al 2003
OsMAPK4/ OsMAPK5	Up-regulated	*Oryza sativa*	Positively regulate salt stress tolerance,	Fu et al. 2002, Xiong et al. 2003
OsMAPK33/ OsMAPK44	Up-regulated	*Oryza sativa*	Play important role in salt stress tolerance through unfavorable ion homeostasis	Lee et al. 2011, Jeong et al. 2006
OsMSRMK2/ OsMSRMK3	Up-regulated	*Oryza sativa*	Play role in salt stress signaling pathway in rice.	Agrawal et al. 2002, 2003
OsmiR396c/ OsmiR393	Up-regulated	*Oryza sativa*	Overexpression shows sensitive phenotype in salt stress	Gao et al. 2010, 2011
OsWRKY45-2	Up-regulated in after 4 day	*Oryza sativa*	Positively regulates ABA signaling and negatively regulates rice response to salt stress.	Tao et al. 2011
OsTIFY11a	Up-regulated	*Oryza sativa*	Regulation seed germination	Ye et al. 2009
OsDhn1	Up-regulated	Oryza sativa	Play important role in enhancing salt and drought stress tolerance	Not reported
OsERF922	Down-regulated	*Oryza sativa*	Negatively regulate the salt stress tolerance	Liu et al. 2012
OsSKIPa	Up-regulated	*Oryza sativa*	Positively regulate salt and drought stress tolerance	Hou et al. 2009
c-GS2	Up-regulated	*Oryza sativa*	Accumulation of glutamine leads to salt stress tolerance	Hoshida et al. 2000

otsA + otsB	Up-regulated	*Escherichia coli*	Positively regulate salt and drought stress tolerance through the accumulation of trehalose	Garg et al. 2002
hva1	Up-regulated	*Hordeum vulgare*	Accumulation of HVA1 leads to salt and drought stress tolerance	Xu et al. 1996
**Salt stress related gene in ABA independent pathway**				
OsDREB1A	Up-regulated	*Oryza sativa*	Regulation of stress-responsive gene expression and accumulation of proline.	Dubouzet et al. 2003
OsDREB1F	Up-regulated	*Oryza sativa*	ABA-responsive regulation of gene expression	Wang et al. 2008
OsDREB2A	Up-regulated	*Oryza sativa*	Regulation of salt stress tolerance	Mallikarjuna et al. 2011
OsCPK12	Not regulated at the transcriptional level in response to salt stress	*Oryza sativa*	An essential positive regulator of tolerance to salt stress.	Asano et al. 2012
OsrbohI / OsAPx2/ OsAPx8	Regulated by *OsCPK12*	*Oryza sativa*	Play important role in H_2O_2 homeostasis	Asano et al. 2012
codA	Up-regulated	*Arthrobacter globiformis*	Positively regulate salt and cold stress tolerance in rice	Sakamoto et al 1998
Mn-SOD	Up-regulated	*Saccharomyces cerevisiae*	Transgenic plants showed enhanced tolerance to salt	Tanaka et al. 1999
katE	Up-regulated	*Escherichia coli*	It decompose H_2O_2 and increase salt stress tolerance	Motohashi et al. 2010
OsGSK1	Down-regulated	*Oryza sativa*	Negatively regulate the salt stress tolerance.	Koh et al. 2007
OsNADP-ME2	Up-regulated	Oryza sativa	Play important role in enhancing tolerance of plants to salt and osmotic stress	Liu et al. 2007
**Genes involved in ionic stress response pathway**				
OsSOS1/ OsCIPK24 / OsCBL4	Up-regulated	*Oryza sativa*	Important in Calcium signaling	Atienza et al. 2007
OsAKT1	Up-regulated	*Oryza sativa*	Play important role in enhancing salt stress tolerance	Golldack et al. 2003
OsCLC1	Up-regulated	*Oryza sativa*	Act as proton-chloride antiporters	Nakamura et al. 2006
