# Supplementary figures and images for: Insights into genomics of salt stress response in rice
Source: Rice (N Y). 2013 Oct 28;6:27. doi: 10.1186/1939-8433-6-27 (PMC4883734; doi:10.1186/1939-8433-6-27)

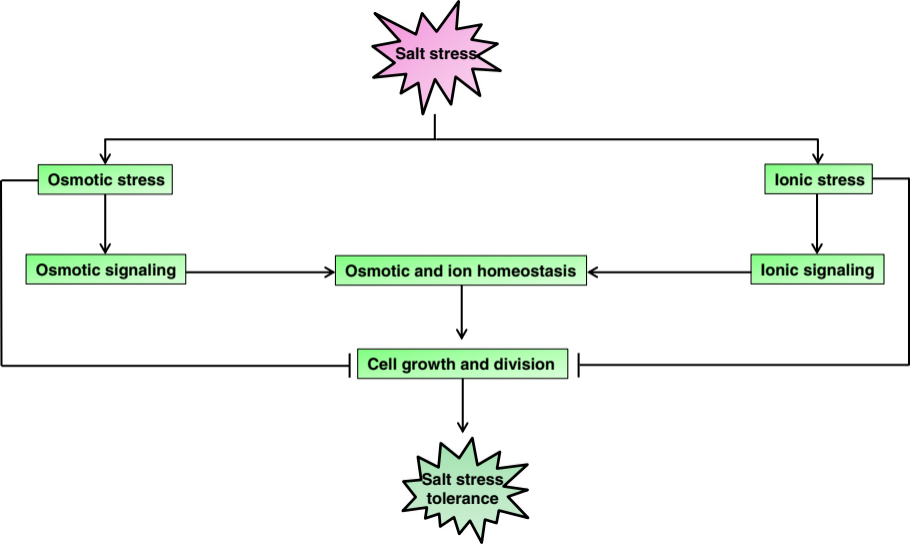

Supplement: Supplementary file 2 — Authors’ original file for figure 1 [file 12284_2013_61_MOESM2_ESM.pdf]

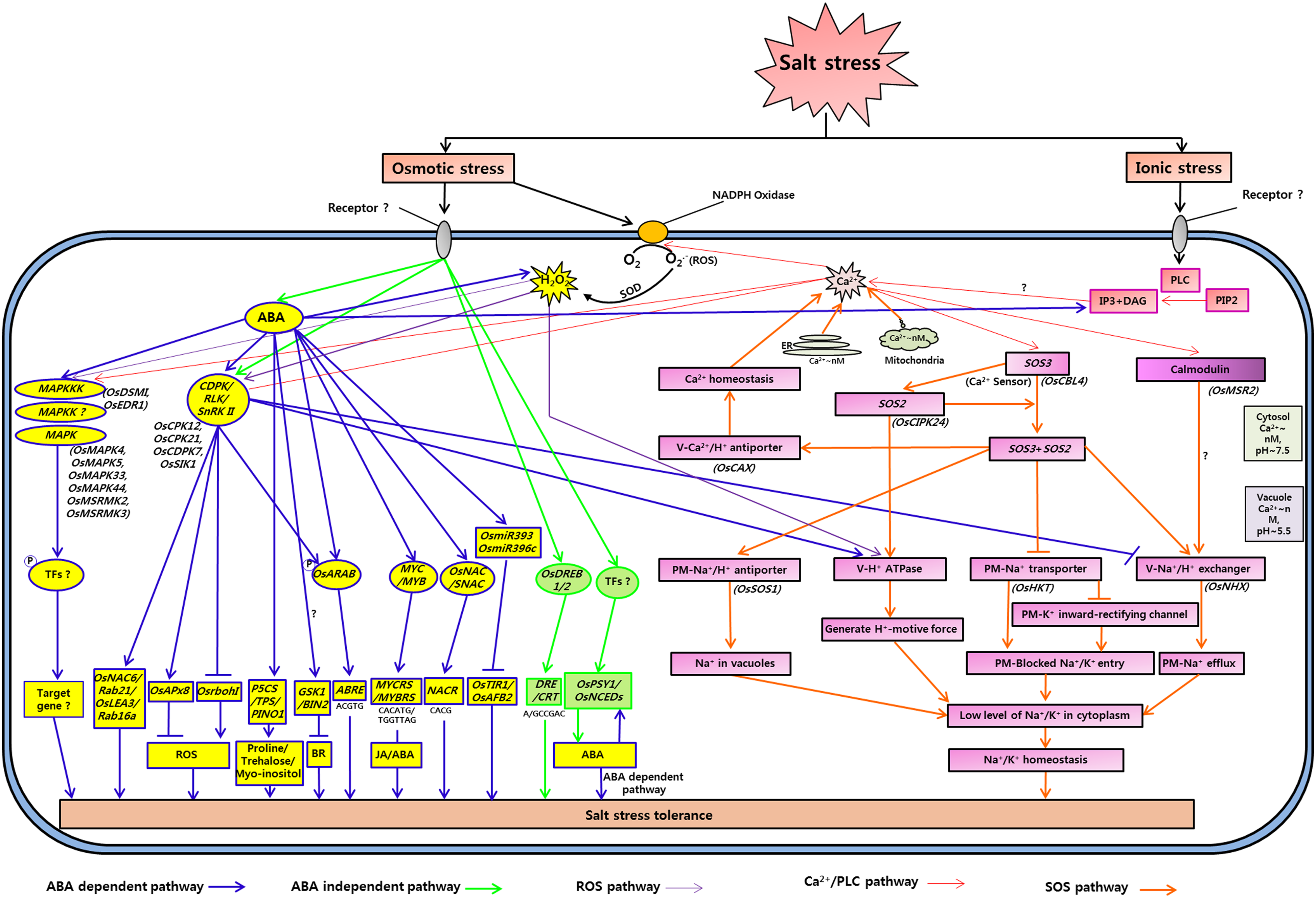

Supplement: Supplementary file 3 — Authors’ original file for figure 2 [file 12284_2013_61_MOESM3_ESM.tiff]

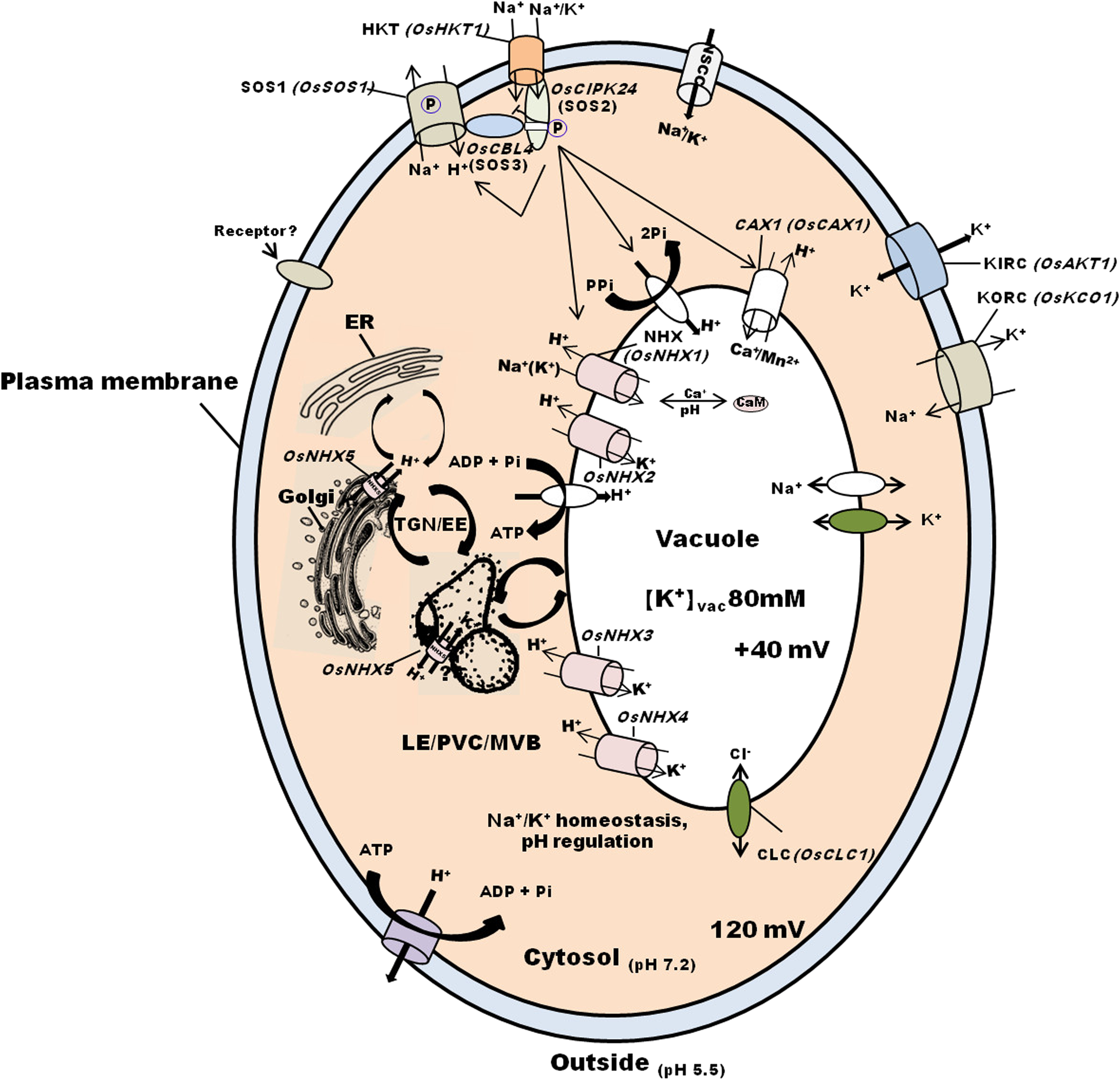

Supplement: Supplementary file 4 — Authors’ original file for figure 3 [file 12284_2013_61_MOESM4_ESM.tiff]
